# Longitudinal evaluation of the impact of standardised packaging and minimum excise tax on tobacco sales and industry revenue in the UK

**DOI:** 10.1136/tobaccocontrol-2019-055387

**Published:** 2020-07-12

**Authors:** Rosemary Hiscock, Nicole H Augustin, J Robert Branston, Anna B Gilmore

**Affiliations:** 1 Tobacco Control Research Group, Department for Health, University of Bath, Bath, UK; 2 Department of Mathematical Sciences, University of Bath, Bath, Somerset, UK; 3 School of Mathematics, University of Edinburgh, Edinburgh, UK; 4 School of Management, University of Bath, Bath, UK

**Keywords:** tobacco industry, packaging and labelling, price, taxation

## Abstract

**Background:**

Standardised packaging for factory-made cigarettes (FM) and roll-your-own tobacco (RYO), and a minimum excise tax (MET) were fully implemented in the UK in May 2017 following a 12-month transition period. This paper is the first to examine effects on tobacco sales volumes and company revenues.

**Methods:**

Analysis of UK commercial supermarket and convenience store electronic point of sale data on tobacco sales. FM and RYO products' data (May 2015–April 2018) yielded 107 572 monthly observations. Expected values from additive mixed modelling were used to calculate trends in: (1) volumes of tobacco sold overall, by cigarette type (FM and RYO) and by seven market segments; and (2) company net revenues. A 10-month period (June 2015–March 2016) before the transition to standardised packs was compared with a 10-month period after the introduction of the MET and full implementation of standardised packs (June 2017–March 2018).

**Results:**

Postimplementation, the average monthly decline in stick sales was 6.4 million (95% CI 0.1 million to 12.7 million) sticks faster than prelegislation, almost doubling the speed of decline. Sales of cheap FM brands, previously increasing, plateaued after implementation. Company monthly net revenues declined from a stable £231 million (95% CI £228 million to £234 million), prelegislation, to £198 million (95% CI £191 million to £206 million) in April 2018.

**Conclusions:**

The concurrent introduction of standardised packaging and MET in the UK was associated with significant decline in sales and in tobacco industry revenues, and the end of the previous growth in cheap cigarette brands that appeal to young and price conscious smokers.

## Introduction

The tobacco industry has been able to keep low priced tobacco available for price-conscious smokers in many countries in order to encourage uptake and discourage quitting yet has continued to grow profits, even where there are rising taxes and declining sales.^1–13^ A growing price gap between cheap and expensive cigarettes driven by the tobacco industry’s pricing strategies is likely to have led to these outcomes.^2 7^ The tobacco industry overshifts taxes on the most expensive cigarettes to maximise profits while undershifting taxes on the cheapest to reduce the impact of tax increases on their retail price.

By mid-2017, the UK government had fully implemented two policies that may address these pricing strategies: standardised packaging^14–16^ for factory-made cigarettes (FM) and roll-your-own tobacco (RYO) and a minimum excise tax (MET) on FM products.^17^ The industry’s ability to create a price gap depends on signalling the premium connotations of its more expensive brands, and conversely indicating that other products are cheap. Standardised packaging threatens its ability to do this by reducing exposure to prosmoking imagery and branding and also increases the salience of health warnings.^18^ This therefore threatens the industry’s profitability model, and likely underpins the tobacco industry’s well-documented intense opposition to the policy.^19 20^

The MET is an addition to existing tobacco taxes: ad valorem tax on FM brands (a tax levied as a percentage of the sales value) and specific tax (tax on volume sold). The MET is an alternative method of calculating the tobacco duty payable on the cheapest FM brands where ad valorem duty might otherwise mean that total duty was lower than a specified minimum (further information on existing tobacco taxes is available elsewhere).^21^ Thus if a product’s price point does not ordinarily incur the level of tax required by the MET, the tobacco company has to pay the higher MET level to the government. In short, the MET effectively creates a de facto minimum price and discourages the selling of cheaper FM products.^22^

A previous descriptive study of linear-only trends in UK sales volumes suggested that FM sales have been falling but RYO sales have been rising in recent years, both prestandardised packaging implementation and during standardised packaging implementation (2013–2018).^23^ The paper’s authors called for a more detailed analysis of the impact of standardised packaging on different market segments to understand the impact on the tobacco industry and its pricing strategies.^23^ This paper’s objective is to examine market segment sales volumes and company revenues surrounding the implementation of standardised packaging and a MET in the UK. The results of this study will be globally important as other jurisdictions consider implementing these policies.

## Methodology

### Data

Nielsen, a global information company, collates electronic point of sales (EPOS) data on tobacco product sales from nearly 90% of UK supermarkets (including a census of sales from stores owned by the largest four UK supermarket chains) and a stratified sample of 15% of convenience stores. Nielsen then scale up collated data to develop sales and pricing estimates for the UK overall and split into 11 geographical areas. Strata for the scaling up are based on Nett Barb TV geography, shop/group type, and fascia (ie, chain affiliation).

Using a hierarchical structure, Nielsen records for each product (or stock keeping unit (SKU)): the tobacco brand, brand family, brand variant,^2^ and specific features of the pack (eg, number of sticks (FM) or weight of contents (RYO), whether packaging is price-marked or (for FM only) standardised, and whether sold as a single or multipack).

For each SKU, 3 years (May 2015–April 2018) of monthly data on volume of sales, sales prices, and extent of distribution of sales within the 11 UK geographical areas were available. For sample design reasons, Nielsen recommend only analysing observations of widely distributed SKU (sold via 10% or more retailers). We therefore excluded monthly observations that did not reach this threshold either in the UK overall or within a given geographical area if sales did not reach the threshold in that area. This left 107 572 monthly observations of widely distributed SKUs, which included 91% of total volume (further sample details are available.^21^)

### Variables

#### Market segment

The tobacco industry splits its products into market segments and has different pricing strategies for each market segment.^2 7^ We allocated brand variants to market segments based on our 2018 comprehensive review of the commercial literature and analysis of Nielsen data. This identified four market segments for FM products (premium, midprice, value, and subvalue) and three for RYO products (premium, midprice, and value). Graphical representation of SKU prices in the prelegislative period (May 2015–April 2016) and an updated review of the commercial literature (January 2015–December 2017) were used to revalidate this allocation. On this basis, three of 348 brand variants were moved into different market segments based on the majority time spent in each market segment.

A few SKUs (5% of RYO and 0.1% of FM by volume) could not be classified by market segment. The majority of these were RYO ‘combi packs’ that included papers and filters within the pack. Unclassified FM products were not present in all months of the data series.

#### Time

Standardised packaging came into force on 20 May 2016 in concert with the EU Tobacco Products Directive.^14 15^ From this date, new packaging was required to be printed in standardised designs but a year-long sell through period was allowed where old branded stock could continue to be sold; thus only from 20 May 2017 were all FM and RYO products required to be sold in standardised packages.^14^ The MET for FM products was introduced on the same day (20 May 2017).^17^ Our dataset, a 36-month period (May 2015–April 2018), provided 1 year of prelegislation data, 1 year of sell through data and 1 year of post full implementation data.

#### Season

Tobacco sales are known to vary by season.^24^ Exploratory analysis of this dataset revealed low sales in January in all 3 years.

#### Volume

We measured volume in terms of the number of sticks of FM and stick-equivalents of RYO sold. RYO stick equivalents were set to a weight of 0.5 g tobacco per stick based on the latest evidence on RYO cigarette size in the UK.^9^ To take into account differing numbers of days per month and so varying opportunities to sell tobacco, we calculated the number of sticks sold per day and then adjusted to a monthly volume (the original scale) by multiplying by the average days per month (365/12).

#### Tobacco industry revenue

Tobacco industry revenues refer to net revenues after tobacco taxes have been paid (ie, net price multiplied by sales), and hence cover the manufacturing, packaging, retailing, and distribution costs with the remainder accruing as industry profit. Revenue was calculated in three stages. First, to allow for the variation in pack size over time,^2^ we calculated price per stick (FM) or stick equivalents (RYO) as our measure of price. To calculate stick equivalent prices for RYO, we again used a weight of 0.5 g per stick.^9^ Real prices were calculated via adjusting nominal prices for inflation to May 2015 prices using the official UK measure of inflation.^25^ Second, we calculated net revenue per stick as the price per stick minus the taxes due on each stick. The taxes were the tobacco taxes (specific, ad valorem, and MET) and VAT.^21^ Third, for each SKU, we multiplied the net price per stick by the volume of sticks sold.

### Statistical analysis

To take account of tobacco industry market segments and non-linear effects of predictor variables including time, and to create confidence intervals in order to understand whether patterns in the data are noise or evidence of a significant change in outcome variables, we estimated additive mixed models (AMM), which is a special case of Generalised Additive Mixed Models. We used R version 3.6.1 with the Mixed GAM Computation Vehicle with Automatic Smoothness (mgcv) package.^26^ Parameter estimation used penalised least squares. We created two models. The dependent variable for the first model was volume and for the second, tobacco industry revenue.

Point of sale data are difficult to model due to a large number of zero sales as products come on and off the market.^27^ We overcame this issue by aggregating the UK sales by market segment and type, that is, summing over SKU for each segment type combination. Thus, in our dataset for modelling volume, rather than each case representing a product/SKU (in a particular month and geographical area), each case represented a market segment (in a month). In our model of tobacco industry revenue, we were interested in the overall impact (rather than by market segment), so in the dataset for modelling, each case represented a month. As with volumes, revenues of the original cases were summed to create the revenue for each month in the dataset used for modelling.

Our dataset for analysis of volume consisted of 292 observations with each observation representing a market segment in a particular month. The sales volume for each case was the summed sales of all SKU sold in all geographies for that market segment and in that month. The independent variables were market segment *m* and two variables related to the observation month (detailed below): time *t* and season *s*. The dependent variable was volume:

$vol_{tm}$, volume of market segment m in month t.

We modelled:

$$vol_{tm}=\alpha +marketsegment_{m}+f_{m}\left(t\right)+f\left(s\right)+e_{tm}$$

assuming a normal distribution for errors $e_{tm}$.

Exploratory analysis showed that the trends of volume in time were non-linear. Hence, we used an AMM, as this is a flexible model, allowing the data to estimate the form of the time trend.^28^

Exploratory assessment of the autocorrelation function (ACF) and partial ACF showed some temporal correlation with volume—the volume sold in 1 month was similar to the volume sold in the previous month. Hence, we assumed an autoregressive process of order 1 (AR1) for the residuals of the model. Ignoring the temporal correlation may lead to a negative bias in the variance estimates and consequently too narrow confidence intervals (CIs).

Model selection (using the Akaike information criterion) showed that the best model (presented in table S1 in the online supplementary file) included smooth functions of time varying by market segment (in other words an interaction between time and segment). The function $f_{m}(t)$ was represented with thin plate regression spline basis.

10.1136/tobaccocontrol-2019-055387.supp1 Supplementary data

The function $f_{m}(t)$ represents any effect of time on volume. Time has 36 months where month 1 is May 2015 and Month 36 is April 2018. Time is therefore a proxy for the different tax events, the introduction of standardised packaging, pricing strategies of the tobacco industry, and any other events that might have impacted volumes (eg, public health campaigns on smoking). Tax events and implementation of standardised packaging happened in the same time period so it is not possible to distinguish between these effects.

The function f(s) represents a seasonal effect for time of the year. Season represents a categorisation of the month of the year where month 1 is May (the first month of the data series) and month 12 is April (the last month of the data series). The effect is represented by a cyclic cubic regression spline, that is, a penalised cubic regression spline whose ends match up to second derivative.

We conducted three sensitivity tests on the effects: first including geography as a dependent variable, second including population size in the model, and third using Nielsen’s UK wide estimates rather than separate estimates for regions/devolved nations (see supplementary file for more details). Model conclusions did not change substantially.

#### Trend estimation using model results

##### Quantity of sticks sold

Estimated mean monthly sales volumes with 95% CIs (overall, by tobacco type and by market segment), across the data series, are presented in plots. CIs were calculated using the model matrix (also called the prediction matrix), estimated model coefficients, and the estimated variance covariance matrix using standard linear model theory.^29^

##### Prelegislation and postlegislation growth or decline

**Figure 1:**
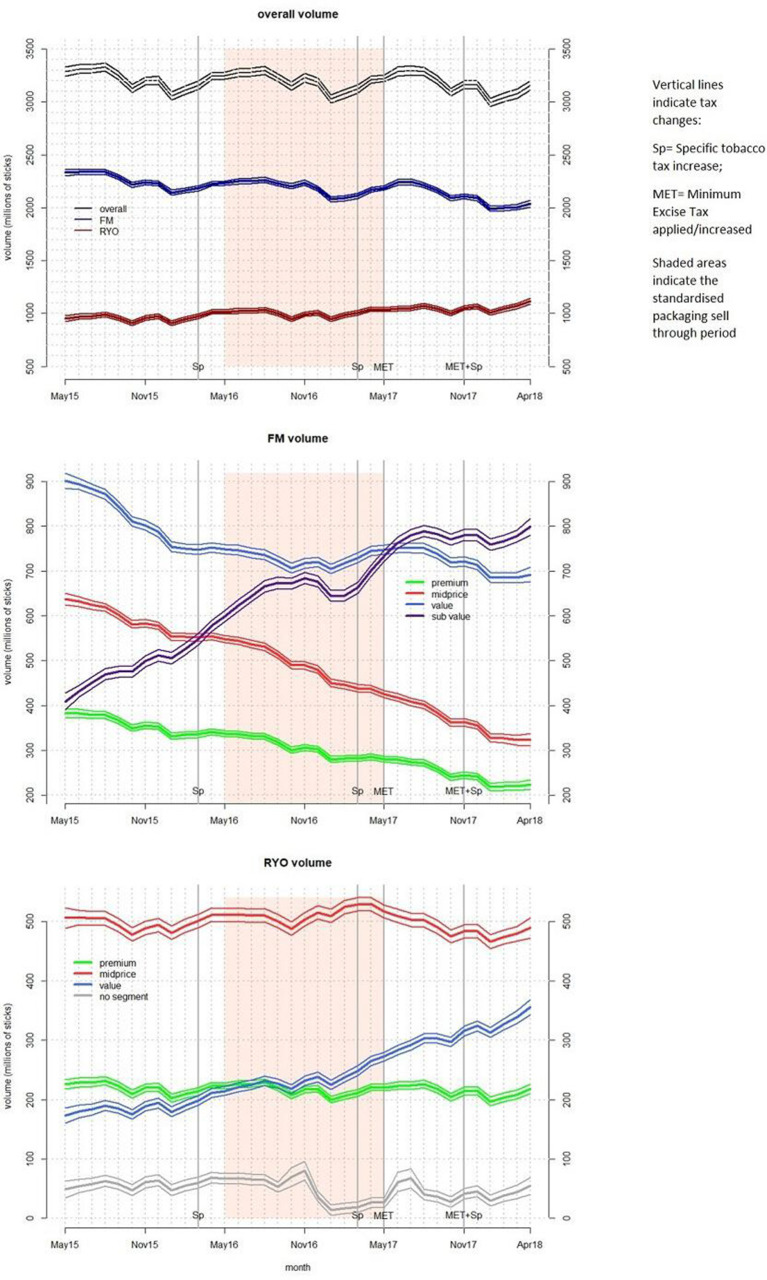
Model-estimated sales volumes (with 95% CIs) over time (A) overall and by product type; (B) for FM products by market segment; and (C) for RYO products by market segment. FM, factory made; RYO, roll your own.

To provide further clarity, we explored whether month-to-month fluctuations in sales volumes (illustrated in figure 1) were on average showing rises or declines in sales prelegislation and postlegislation (overall and by tobacco type). We compared mean monthly change in sales volumes (monthly change was calculated by subtracting modelled previous month monthly sales (in sticks) from modelled sales) over two time periods.

The first time period was 10 months before the sell through period, and the second time period was the same 10 calendar months after the introduction of standardised packaging and MET. The same months (June–March) were used to reduce the impact of seasonal variations. These 10 months did not include the month immediately before the implementation or the month of implementation because changes might already have been underway.

The 95% CIs were also estimated using the model matrix, the estimated model coefficients and the estimated variance covariance matrix.^29^ The estimated model coefficients vector was multiplied with a linear combination of the model matrix for the point estimates, and their standard errors were obtained by premultiplying and postmultiplying the covariance matrix with this linear combination of the model matrix.

We also calculated the difference between mean stick sales within the two periods. If CIs did not cross zero, then a significant change had occurred.

#### Tobacco industry revenue model

Our dataset for revenue consisted of 36 observations with each observation representing a month. We modelled monthly revenue ${rev}_{t}$ for each month time t:

$$\begin{array}{l} rev_{t}=\alpha +f\left(t\right)+\epsilon_{t} \end{array}$$

where the function f(t) is a non-linear effect for month (see online supplementary table 2). This model (table S2 in the online supplementary file) assumes normal errors.

##### Trend estimation using model results

Trends and CIs of overall revenue over time were estimated using similar procedures to those used for the volume model. Our estimates were verified against revenue estimates from other sources^3 30^ and found to be consistent. We found little change in revenue for the preimplementation period (May 2015–May 2016; see table S3 in the online supplementary file), so we estimated mean monthly revenue and CI for this period as a summary statistic.

### Patient and public involvement

We led a session on standardised packaging policy with the UK Centre for Tobacco and Alcohol Studies University of Nottingham panel of smokers and former smokers (now the Tobacco & Nicotine Discussion Group) in October 2017. The group were asked to discuss their thoughts on and experiences of standardised packaging and whether they had noticed any changes to tobacco products and packs both in the sell-off period and thereafter. These discussions helped inform our research questions but were not formally part of our data analysis.

## Results

Sales volumes overall declined over the study period (figure 1a) from 3.29 billion sticks (95% CI 3.24 billion to 3.33 billion) in May 2015 to 3.16 billion (95% CI 3.11 billion to 3.20 billion) in April 2018. FM sales fell from 2.33 billion (95% CI 2.30 billion to 2.36 billion) to 2.04 billion (95% CI 2.01 billion to 2.07 billion), whereas RYO sales grew from 0.95 billion (95% CI 0.93 billion to 0.98 billion) to 1.12 billion (95% CI 1.09 billion to 1.15 billion).

To understand any impact of the legislation, we compare the monthly change in stick sales preimplementation and postimplementation (table 1). Overall, average monthly stick sales were lower than the previous month in both periods. The rate of decline almost doubled postlegislation. On average, the monthly decline was an extra 6.4 million (95% CI 0.1 million to 12.7 million) sticks postlegislation compared with prelegislation. FM sticks were also declining significantly faster postlegislation (extra monthly decline 9.0 million sticks (95% CI 4.3 million to 13.6 million)). RYO sales were rising in both periods, and there was not significant change in the rate of growth.

**Table 1:** Change in stick sales (millions) pre and post standardised packs and MET implementation

	Average monthly change in sticks sales (millions) and 95% CI					
	Period 1: preimplementation June 2015–March 2016		Period 2: post full implementation June 2017–March 2018		Difference between post and pre	
Overall	−**7.0**	(−12.4 to −1.6)	−**13.3**	(−19.5 to −7.2)	−**6.4**	(−12.7 to −0.1)
FM	−**11.5**	(−15.0 to −8.0)	−**20.4**	(−24.5 to −16.3)	−**9.0**	(−13.6 to −4.3)
RYO	**4.5**	(1.3 to 7.6)	**7.1**	(3.6 to 10.5)	**2.6**	(−1.3 to 6.5)

FM, factory made; RYO, roll your own.

Monthly sales volumes of FM premium, midprice, and value products fell significantly through the study period (figure 1b). FM premium monthly sales declined from 383 million (95% CI 373 million to 393 million) sticks in May 2015 to 224 million (95% CI 213 million to 234 million) in April 2018, FM midprice sales fell from 637 million (95% CI 624 million to 651 million) to 324 million (95% CI 310 million to 337 million), and FM value sales fell from 902 million (95% CI 885 million to 918 million) to 692 million (95% CI 676 million to 709 million).

FM subvalue sales, however, increased significantly from 409 million (95% C I 391 million to 427 million) to 780 million (95% CI 767 million to 793 million) in July 2017 (just after full implementation of legislation), and did not increase significantly afterwards. Sales were 799 million (780 million to 817 million) in April 2018.

There was no significant change in sales of RYO premium and midprice products during the study period (figure 1c). RYO value sales grew significantly from 174 million (95% CI 161 million to 186 million) to 355 million (95% CI 343 million to 368 million).

### Tobacco industry revenue

**Figure 2:**
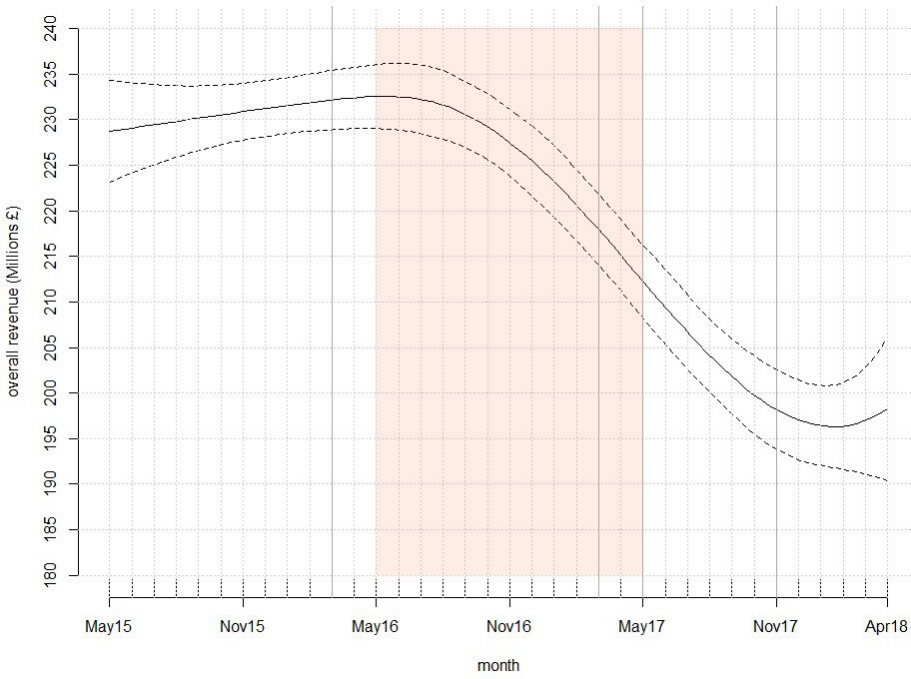
Model-estimated tobacco industry net revenue (and 95% CIs) over time.

Tobacco industry monthly revenue was stable until the beginning of the sell through period (figure 2 and table S3 in the online supplementary file). Thenceforth, revenue declined steadily throughout the sell through period and post full implementation of standardised packaging.

Prelegislation, tobacco industry mean monthly net revenue was estimated to be £231 million (95% CI £228 million to £234 million) and did not vary significantly. During the sell through period, revenue declined from £233 million (£229 million to £236 million) in May 2016 to £212 million (95% CI £208 million to £216 million) in May 2017. By April 2018 (the final month of the data series), revenue had declined to £198 million (£191 million to £206 million). Thus, monthly net revenue was estimated to have declined by 13% (or £31 million) over the study period.

## Discussion

Standardised packaging was fully introduced concurrently with a MET in May 2017. After implementation, overall tobacco sales declined faster as did sales of FM products. There was no significant change in the growth of RYO products.

The cheapest FM cigarettes (FM subvalue brands) stopped growing post MET/standardised packaging. Our previous pricing analysis^21^ suggests that FM subvalue prices rose around the introduction of the MET such that they were then similar to FM value product prices. Although sales of the cheapest RYO products rose, the overall decline in sales implies that downtrading to the cheapest RYO did not (entirely) substitute for the disappearance of cheap FM brands. Thus, options for price conscious smokers to continue to smoke via purchasing cheap products have been limited, which has implications for improving public health and reducing health inequalities.^31 32^ There is no evidence that such smokers have turned to illicit tobacco as UK government (Her Majesty's Revenue and Customs (HMRC)) estimates for illicit tobacco have changed little over this period.^33^

The tobacco industry claimed that sales in Australia, the first country to introduce standardised packaging, may have risen in the short term.^34^ Our research concurs with Australian peer-reviewed research suggesting that post standardised packaging and a tax rise consumption overall fell.^35^

An evaluation of the introduction of a MET on Spanish cigarettes found no decline in male prevalence and a small long-term, but no short term, decline in female prevalence.^36^ The lack of effect was thought to be due to switching to cheaper RYO tobacco, on which no MET had been applied. In the UK, the simultaneous introduction of standardised packaging with the MET, which mandates a RYO minimum pack size of 30 g^14^ (a considerable increase on the previously most popular 12.5 g pack^2^), may have made switching from FM to RYO less attractive in the UK. Furthermore, there were concurrent tax changes during this period that markedly increased taxes on RYO products.^21^ However, these changes have not yet been sufficient to lead to sales decline of RYO, and hence, there remains considerable scope to increase RYO taxes further.

The introduction of standardised packaging in May 2016 coincided with a decline in tobacco industry revenues. This would imply that standardised packaging has reduced revenue from the sale of an addictive deadly substance^37^ and signals why the industry has been so opposed to plain packaging.

### Strengths and limitations

The strengths of this paper are that we were able to evaluate a policy, standardised packaging, which was brought in gradually because our GAM modelling meant we were not restricted to a simple before-and-after comparison of volume trends. We were simultaneously able to monitor impacts of tax changes, including the introduction of a MET, on volumes.

Given the late appearance of standardised packs in the implementation period,^21^ it is not possible to distinguish statistically between effects of standardised packaging, minimum pack sizes, and the four taxation changes in 2016 and 2017. Thus, our analysis generally focused on differences at the beginning and end of the analysis period rather than before and after each policy change.

Nielsen use EPOS data to provide estimates of the UK tobacco market. The advantage of Nielsen data is that it is based on a census of sales at stores owned by the big four UK supermarkets. Nielsen estimates that 70%–80% tobacco sales are from convenience stores where Nielsen estimates are based on a sample. However, Nielsen estimates of the UK entire grocery market lie within estimates from other sources^38 39^ suggesting they are reasonable. It should, however, be noted that our dataset did not cover all tobacco-related sales. For example, it did not include sales of: RYO rolling papers and filters (unless sold within tobacco pouches); pipe tobacco; cigars and cigarillos; and sales from specialist tobacconists. Tobacconists revenue is only £454 million per year (under 2% of total FM plus RYO sales) and has declined by 9% (2013–2018), pipe tobacco is now about 0.6% of total tobacco sales, and sales of cigars and cigarillos (barely covered by standardised packaging legislation and representing 0.8% of total tobacco sales)^40^ were thought not to be growing in response to standardised packaging during the study period.^41^

Nielsen has not provided details of how they scale up the sample data to the population level, and hence, for example, whether they use modelling. They do, however, suggest excluding low distributed products for sampling reasons. This has meant our analysis covers only about 90% of the UK cigarette market and hence that our estimates of volume and revenue are likely to be conservative. Nevertheless, they should offer reasonable insights into the changing nature of sales trends since our market coverage does not materially change during the period of analysis.

Tobacco sales volumes may also have been affected by other changes in this period. For example, Euromonitor data suggest that the value of sales of vaping products (eg, e-cigarettes) grew,^42^ although official sources suggest that prevalence of vaping in Great Britain was stable and under 6%.^43^ UK population growth during the study period (1.3 million people (0.2%))^44^ may have had an impact on tobacco sales so small reductions in sales volumes translate to slightly larger reductions in terms of *per capita* consumption. Furthermore, some disadvantaged smokers (smokers are more disadvantaged than the general population) were having incomes squeezed by benefit freezes, benefit payment delays with the introduction of universal credit (a new income benefit), and growth in housing private rental costs.^45 46^

Sales data provide information on products rather than smokers who purchase the product. Thus, we are unable to assess the extent to which reduced volumes were the result of smokers quitting as opposed to reducing consumption. Moreover, although we have described trends, and changes in trends, we cannot prove any changes were the direct result of the tobacco control legislation introduced.

### Conclusions

Introducing standardised packaging and a MET was associated with a decline in tobacco sales and tobacco industry revenue. The introduction of the MET coincided with the end of sales growth in FM brands that had previously been cheapest. We therefore recommend other countries consider implementing both policies for the improvement of public health.

**What this paper adds**

- Standardised packaging was first enacted in Australia in December 2012. In the UK, standardised packaging legislation was enacted in May 2016 and was fully in place in May 2017, when a minimum excise tax (MET) was implemented.
- Previous research on the impacts of standardised packaging and MET is limited: in Australia, comprehensive sales data are not available to public health researchers, but available data suggested volumes fell after standardised packaging was introduced and smokers downtraded to cheaper brands. In the UK, a descriptive study suggested that factory-made (FM) cigarette volumes were lower and roll-your-own (RYO) tobacco volumes were higher in 2018, poststandardised packaging, than 5 years previously. In Spain, a MET in 2006 led to downtrading from FM to RYO.
- This paper is the first to examine the impact of UK standardised packaging and the MET on tobacco market segment sales and tobacco industry net revenue.
- The analysis in this paper showed that post full implementation of standardised packaging and the MET, overall tobacco sales declined faster. Continuing growth in RYO was not able to compensate for accelerating decline in FM; cheap FM brands stopped growing. Tobacco industry revenue, which was stable prestandardised packaging legislation, declined thereafter.
- The concurrent introduction of standardised packaging and MET can lead to significant declines in tobacco sales and in tobacco industry revenues while also reducing sales of cheap cigarette that appeal to young and price conscious smokers.

## Data Availability

Data may be obtained from a third party and are not publicly available. We would like to thank Nielsen and Public Health England for provision of the sales data. Our contractual agreement does not allow us to share data.
