# Supplementary material for: Longitudinal evaluation of the impact of standardised packaging and minimum excise tax on tobacco sales and industry revenue in the UK
Source: Tob Control. 2020 Jul 12;30(5):515–22. doi: 10.1136/tobaccocontrol-2019-055387 (PMC8394752; doi:10.1136/tobaccocontrol-2019-055387)
Supplement: Supplementary data [file tobaccocontrol-2019-055387supp001.pdf]

Supplementary material

Supplementary tables

Table S1: Volume model details

Formula: standardised_monthly_volume ~ s(time, by = mktseg, k = 20, bs = "tp") + s(season, bs = "cc", k = 11) + mktseg
Family: Gaussian Link function: identity
R-sq.(adj)=0.998 Deviance explained=99.8% fREML=4959.2 Scale est.=114.16 trillion N=292[1]
Parametric coefficients:

		Estimate	Std. Error	t value	p	
	(Intercept)	303965746	1824587	166.59	<.001	***
Market segment						
	FM premium	0 (REF)				
	FM midprice	178961670	2494396	71.75	<.001	***
	FM value	450816692	2496121	180.61	<.001	***
	FM subvalue	336326426	2496162	134.74	<.001	***
	RYO premium	-87026795	2489433	-34.96	<.001	***
	RYO midprice	195173312	2497612	78.14	<.001	***
	RYO value	-60816524	2505029	-24.28	<.001	***
	no segment	-258484540	2447506	-105.61	<.001	***

Approximate significance of smooth terms:

	edf	Ref.df	f	p	
FM premium	2.2	2.7	260.0	<.001	***
FM midprice	4.3	5.3	537.9	<.001	***
FM value	8.3	10.2	90.7	<.001	***
FM subvalue	12.1	14.5	319.4	<.001	***
RYO premium	1.0	1.0	1.7	.197	
RYO midprice	9.3	11.5	5.5	<.001	***
RYO value	3.8	4.7	195.8	<.001	***
no segment	5.5	6.9	10.2	<.001	***
month	8.1	9.0	13.7	<.001	***

Signif. codes: 0 '***' 0.001 '**' 0.01 '*' 0.05 '.' 0.1 ' ' 1

[1]There were 4 more cases than expected from 36 months*8 market segments because case aggregation by tobacco type split the no segment into FM and RYO no segment and FM unclassified products were not available in all months

Hiscock R, *et al. Tob Control* 2021; 30:515–522. doi: 10.1136/tobaccocontrol-2019-055387

Table S2: Tobacco industry revenue model details

Formula: revenue ~ s(time, bs = " cr", k = 10)

Family: Gaussian Link function: identity
R-sq.(adj)= 0.848 Deviance explained=86.6% REML = 584.15 Scale est.= 34.365 billion n=36

Parametric coefficients:

	Estimate	Std. Error	t value	p	
(Intercept)	219549242	943716	232.6	<.001	***

Approximate significance of smooth terms:

	edf	Ref.df	f	p	
Time	3.827	4.479	47.53	<.001	***

Signif. codes: 0 '***' 0.001 '**' 0.01 '*' 0.05 '.' 0.1 ' ' 1

Hiscock R, *et al. Tob Control* 2021; 30:515–522. doi: 10.1136/tobaccocontrol-2019-055387

Table S3 Modelled tobacco industry revenues (sales values net of taxes)

Date	Tobacco industry Revenue (modelled)
Pre legislation	
May-15	229M (223M to 234M)
Jun-15	229M (224M to 234M)
Jul-15	229M (225M to 234M)
Aug-15	230M (226M to 234M)
Sep-15	230M (227M to 234M)
Oct-15	231M (227M to 234M)
Nov-15	231M (228M to 234M)
Dec-15	231M (228M to 234M)
Jan-16	232M (229M to 235M)
Feb-16	232M (229M to 235M)
Mar-16	232M (229M to 235M)
Apr-16	232M (229M to 236M)
May-16	233M (229M to 236M)
Sell through (from 20th May): New packaging must be standardised but old stock may be sold	
Jun-16	233M (229M to 236M)
Jul-16	232M (229M to 236M)
Aug-16	232M (228M to 235M)
Sep-16	231M (227M to 234M)
Oct-16	229M (226M to 233M)
Nov-16	228M (224M to 231M)
Dec-16	225M (222M to 229M)
Jan-17	223M (219M to 227M)
Feb-17	221M (217M to 224M)
Mar-17	218M (214M to 222M)
Apr-17	215M (211M to 219M)
May-17	212M (208M to 216M)
Full implementation (from 20th May): All FM and RYO must be sold in standardised packs	
Jun-17	209M (205M to 213M)
Jul-17	207M (203M to 211M)
Aug-17	204M (200M to 208M)
Sep-17	202M (198M to 206M)
Oct-17	200M (196M to 204M)
Nov-17	198M (194M to 203M)
Dec-17	197M (193M to 201M)
Jan-18	196M (192M to 201M)
Feb-18	196M (192M to 201M)
Mar-18	197M (191M to 203M)
Apr-18	198M (191M to 206M)

Hiscock R, *et al. Tob Control* 2021; 30:515–522. doi: 10.1136/tobaccocontrol-2019-055387

Sensitivity tests

Geography

Initially we included geography in our modelling as this allowed us to estimate the effect of geography, to check whether time trends differed by geography and to assess whether geography is a confounder for the time effect. Exploration of this volume model (table S4) showed that the estimated time effects did not differ between geography significantly and overall time trends by segment did not change whether geography was included in the model or not. Hence, although geography had a large amount of explanatory power, with large differences in volume between segments sold in the different geographies we concluded that it was not a confounder variable. This allowed us to aggregate over geography in the model presented in the main manuscript. Including geography required a model with a more complex Tweedie distribution. Our final model reported in the main paper was simpler, more parsimonious and fitted the data better.

Table S4: Volume model details with geography

Formula: standardised_monthly_volume ~ s(time, by = mktseg, k = 20, bs = "ad") + s(season, bs = "cc", k = 11) + geography + mktseg
Family: Tweedie(p=1.) Link function: log
R-sq.(adj)=0.888 Deviance explained=89.6% fREML=14368 Scale est.=6988.4 n=3176[1]

Parametric coefficients:

		Estimate[2]	Std. Error	t value	Pr(>|t|)	
	(Intercept)	17.61	0.09	184.972	<.001	***
Geography						
	Central England	0 (REF)				
	East of England	-.64	.10	-6.270	<.001	***
	Lancs and English Border	-.20	.09	-2.097	.036	**
	London	.29	.09	3.385	.001	**
	North East	-1.20	.12	-10.384	<.001	***
	South & South East	-.30	.10	-3.198	.001	***
	South West	-1.41	.12	-11.655	<.001	***
	Wales & West	-.48	.10	-4.828	<.001	***
	Yorkshire	-.43	.10	-4.374	<.001	***
	Northern Ireland	-1.61	.13	-12.662	<.001	***
	Scotland	-.34	.10	-3.502	<.001	***
Market segment						
	FM premium	0 (REF)				
	FM midprice	.50	.10	5.117	<.001	***
	FM value	.99	.09	10.921	<.001	***
	FM subvalue	.78	.09	8.288	<.001	***
	RYO premium	-.26	.11	-2.400	.016	**
	RYO midprice	.53	.09	5.634	<.001	***
	RYO value	-.19	.11	-1.807	.071	**
	no segment	-1.82	.13	-14.103	<.001	***

Approximate significance of smooth terms:

	edf	Ref.df	f	p	
FM premium	1.41	1.75	12.910	<.001	***
FM midprice	2.22	2.94	17.988	<.001	***
FM value	2.02	2.54	5.314	<.001	**
FM subvalue	5.41	6.37	15.137	<.001	***
RYO premium	1.00	1.00	0.986	0.321	
RYO midprice	1.00	1.00	0.181	0.670	
RYO value	1.00	1.00	41.087	<.001	***
no segment	11.26	12.19	28.883	<.001	***
	8.86	9.00	40.669	<.001	***

Signif. codes: 0 '***' 0.001 '**' 0.01 '*' 0.05 '.' 0.1 ' ' 1

[1]There were 8 more cases than expected from 36 months*11geographies*8 market segments because case aggregation by tobacco type split the no segment into FM and RYO no segments

[2]The exponentiated model coefficients can be interpreted as the expected proportion difference in volume for a unit increase in the covariate e.g. the coefficient for East of England is -0.64. This means that East of England has exp(-0.64)= 0.53 of the volume of Central England (the reference category). In other words East of England has 47% less volume than Central England.

Population size

It was not possible to adjust for population size by including it as an explanatory variable due to the almost perfect correlation of population size with time. Hence in order to take account of the growing population size we also fitted a model where the dependent variable was volume per capita $volpercap_{tm} = vol_{tm}/pop_t$, with volume ($vol$) in month t and population size (pop_t) at month t. Population size was interpolated between mid-year ONS estimates to yield monthly figures. The conclusions of this model were the same as for the model presented in the main manuscript. We did not present this volume per capita model in our main manuscript because such models help us understand consumption trends rather than sales trends which relate to company profitability.

Nielsen UK estimates

Nielsen provides data as UK wide estimates and also estimates for each region/devolved nation separately. We conducted sensitivity analysis using four Nielsen datasets:

a) Aggregation of 11 geographical datasets of regions and devolved nations excluding products distributed to <10% stores (the model presented in the main manuscript),

b) Aggregation of 11 geographical datasets of regions and devolved nations including all products

c) UK wide dataset excluding products distributed to <10% stores

d) UK wide dataset including all products

Analyses a, b and d reached similar conclusions. Our use of analysis (a) as our model in the main manuscript is supported by our use of this dataset for the revenue analysis in this paper and our analysis of price changes.[1]

1. Hiscock R, Augustin N, Branston J, et al. Standardised packaging, Minimum Excise Tax, and RYO focussed tax rise implications for UK tobacco pricing. *PloS one* 2020 doi: https://doi.org/10.1371/journal.pone.0228069
